# KnowVolution of the Polymer-Binding Peptide LCI for Improved Polypropylene Binding

**DOI:** 10.3390/polym10040423

**Published:** 2018-04-10

**Authors:** Kristin Rübsam, Mehdi D. Davari, Felix Jakob, Ulrich Schwaneberg

**Affiliations:** 1 Institute of Biotechnology, RWTH Aachen University, Worringerweg 3, D-52074 Aachen, Germany; k.ruebsam@biotec.rwth-aachen.de (K.R.); m.davari@biotec.rwth-aachen.de (M.D.D.); 2 DWI—Leibniz-Institute for Interactive Materials, Forckenbeckstrasse 50, D-52074 Aachen, Germany; jakob@dwi.rwth-aachen.de

**Keywords:** polymer-binding peptides, anchor peptides, directed evolution, surface functionalization, immobilization

## Abstract

The functionalization of polymer surfaces by polymer-binding peptides offers tremendous opportunities for directed immobilization of enzymes, bioactive peptides, and antigens. The application of polymer-binding peptides as adhesion promoters requires reliable and stable binding under process conditions. Molecular modes of interactions between material surfaces, peptides, and solvent are often not understood to an extent that enables (semi-) rational design of polymer-binding peptides, hindering the full exploitation of their potential. Knowledge-gaining directed evolution (KnowVolution) is an efficient protein engineering strategy that facilitates tailoring protein properties to application demands through a combination of directed evolution and computational guided protein design. A single round of KnowVolution was performed to gain molecular insights into liquid chromatography peak I peptide, 47 aa (LCI)-binding to polypropylene (PP) in the presence of the competing surfactant Triton X-100. KnowVolution yielded a total of 8 key positions (D19, S27, Y29, D31, G35, I40, E42, and D45), which govern PP-binding in the presence of Triton X-100. The recombination of two of the identified amino acid substitutions (Y29R and G35R; variant KR-2) yielded a 5.4 ± 0.5-fold stronger PP-binding peptide compared to LCI WT in the presence of Triton X-100 (1 mM). The LCI variant KR-2 shows a maximum binding capacity of 8.8 ± 0.1 pmol/cm^2^ on PP in the presence of Triton X-100 (up to 1 mM). The KnowVolution approach enables the development of polymer-binding peptides, which efficiently coat and functionalize PP surfaces and withstand surfactant concentrations that are commonly used, such as in household detergents.

## 1. Introduction

Protein-based adhesion promoters enable directed protein and peptide immobilization onto material surfaces with a broad range of applications in enzyme catalysis, anti-microbial coatings, drug delivery systems, and biosensors [1,2,3,4]. The class of polymer-binding peptides (PBPs) are mainly used in surface functionalization at ambient temperature, in aqueous solutions under mild and environmentally-friendly conditions [5,6].

PBPs were found to specifically recognize and bind to polymer surfaces via π–π interactions, hydrophobic and electrostatic interactions, and hydrogen bonding [7,8,9,10]. PBP binding depends on the peptide conformation, the amino acid content and the chemical composition, the molecular structure, the size, and the shape of the polymer [11]. For instance, peptides that bind to polystyrene were reported to be enriched in aromatic amino acids, carrying motifs such as WXXW (X represents any amino acid and W represents tryptophan) [12] and/or containing phenylalanine, tyrosine, or tryptophan at the *N*-terminus of the peptide [13]. The binding mechanism of these aromatic amino acids and the aromatic moieties of polystyrene is primarily based upon the stacking of aromatic ring systems [13,14]. In contrast to this, poly(methyl methacrylate) (PMMA)-binding peptide c2 was found to rely on the C-terminal proline residue resulting in a conformation capable of forming hydrogen bonding to ester groups in PMMA [14].

PBPs have been identified for chemically diverse polymers, such as PMMA [7], chlorine-doped polypyrrole (PPyCl) [5], poly(l-lactide) (PLLA) [15], polystyrene [16,17], and polypropylene (PP) [18,19]. PP is one of the most widespread commercial polymers with numerous applications in the textile, packaging, or biomedical industry and was therefore selected for understanding molecular interactions with our selected PBP liquid chromatography peak I peptide, 47 aa (LCI). Unmodified PP surfaces are hydrophobic (water contact angel 103°; no functional group for electrostatic interactions, or aromatic residues for π–π interactions), and interactions with peptides and surfactants are mainly governed by hydrophobic interactions.

Binding peptides can be directly selected from phage display libraries [14] or cell surface display libraries [20]. The binding peptides are often 7–12 amino acids in size [21], and the introduction of non-natural amino acids offers attractive opportunities for binding (e.g., bipyridylalanine for metal-binding peptides) [22]. A conceptually different approach that is especially useful for peptides, which are >30 amino acids, is the reported Peptide–Polymer evolution protocol (PePevo) [17], which employs a tailor-made polymerase with up to 60 mutations per 1000 bps. The PP-binding peptide LCI (originating from *Bacillus subtilis* [23]) was improved in PP-binding strength through one round of directed evolution to validate the PePevo [17].

The non-ionic surfactant Triton X-100 consists of a polydisperse preparation of *p*-(1,1,3,3-tetramethylbutyl) phenyl) poly (oxyethylene) with 10 oxyethylene units per molecule on average [24]. Nonionic surfactants such as Triton X-100 interact with proteins through hydrophobic interactions and are therefore often tolerated by proteins [25].

It is reported that peptide adsorption on a hydrophobic surface results from an interplay of solvation, surface, and intra-peptide forces [26]. In a recent joint experimental and simulation study, Horinek et al. revealed that the adsorption of a mildly hydrophobic peptide from water to a solid hydrophobic surface is governed by a complex interplay of opposing interactions that largely compensate each other and that there is no single mechanism explaining the hydrophobic attraction between the peptide and the surface [26]. Modes of interactions between the PBP and polymer surfaces are often not well-understood [27].

Knowledge-gaining directed evolution (KnowVolution) enables the tailoring of peptide-binding strength, while simultaneously generating knowledge about each PBP–surface interaction. A KnowVolution campaign is performed in four phases: in phase (I) potential key positions are identified, in phase (II) beneficial substitutions at identified positions are determined, in phase (III) amino acid substitutions are rationally selected by computational assisted analysis, and finally in phase (IV) beneficial substitutions are efficiently recombined [28]. The KnowVolution strategy has already been successfully applied for several enzymes, for instance to increase the resistance of cellulase (CelA2) to ionic liquids and deep-eutectic solvents [29], to increase the activity of alkaline protease (BgAP) at low temperatures [30], or to reduce the oxygen dependency of a glucose oxidase (GOx) [27].

Here, we report the first KnowVolution campaign to improve the binding strength of a small peptide, adhesion promoter LCI. LCI’s binding strength was increased toward PP in the presence of a non-ionic surfactant (Triton X-100). In total, 1044 LCI variants were screened in a 96-well microtiter plate (MTP) format for increased binding, and computational-assisted analysis of the identified substitutions yielded molecular insights into the LCI–PP-binding modes; finally, a recombination variant with significantly increased binding strength was identified.

## 2. Materials and Methods

All the chemicals used were analytical-reagent grade or higher and were purchased from Sigma-Aldrich Corp. (St. Louis, MO, USA), AppliChem GmbH (Darmstadt, Germany), and Carl Roth GmbH (Karlsruhe, Germany). Oligonucleotides were acquired from Eurofins MWG Operon (Ebersberg, Germany) in salt-free form. Enzymes were obtained from New England Biolabs GmbH (Frankfurt am Main, Germany). Plasmid extraction and PCR purification kits were purchased from Macherey-Nagel GmbH & Co. KG (Düren, Germany) and Qiagen (Hilden, Germany). The BCA protein assay kit was obtained from Novagen EMD Chemicals Inc. (San Diego, CA, USA). Black polypropylene microtiter plates (MTP) were obtained from Greiner Bio-One GmbH (Frickenhausen, Germany). Plasmid pET28a(+) (Novagen, Darmstadt, Germany) was used as expression vector. The *E. coli* strains DH5α and BL21-Gold (DE3) were purchased from Agilent Technologies (Santa Clara, CA, USA). *E. coli* DH5α was used as cloning host and *E. coli* BL21-Gold (DE3) was used as protein expression system.

### 2.1. Library Generation

The generation of the wildtype construct pET28a::EGFP-10xAla-TEV-Cys-LCI and negative control pET28a::EGFP-10xAla-TEV was performed as previously described [19]. Random mutagenesis was performed as described earlier to generate an epLCI library [17].

Site-saturation mutagenesis (SSM) libraries were generated as described by Wang et al. [31]. The SSM libraries were constructed using primers containing NNK codons for randomization (20 μM each, see Table S1 for the 26 SSM primer sequences). PfuS DNA polymerase (2.5 U) was mixed with dNTPs (10 mM), template (0.4 ng/μL), forward, and reverse primers (20 µM each). Site-saturation mutagenesis was performed in two stages (first stage: 98 °C for 30 s; one cycle, 98 °C for 15 s, 55–65 °C for 30 s, 72 °C for 3 min; 5 cycles and second stage: 98 °C for 15 s; 55–65 °C for 30 s, 72 °C for 3 min; 25 cycles; 72 °C for 4 min; one cycle). The parental DNA was digested (20 U *Dpn*I, 16 h, 37 °C), and the PCR product was purified (PCR clean-up gel extraction kit, Macherey-Nagel) and transformed into *E. coli* BL21-Gold (DE3) for expression.

### 2.2. Site-Directed Mutagenesis

Site-directed mutagenesis (SDM) was performed at positions Y29 and G35 as described by Wang et al. [31]. PCR was performed with F-Y29R in combination with R-Y29R primer and with F-G35V in combination with R-G35V primer (20 μM each, see Table S1 for primer sequences). PfuS DNA polymerase (2.5 U) was mixed with dNTPs (10 mM), template (0.4 ng/μL), forward, and reverse primers (20 µM each). Site-saturation mutagenesis was performed in two stages (first stage: 98 °C for 30 s; one cycle, 98 °C for 15 s, 55–65 °C for 30 s, 72 °C for 3 min; 5 cycles and second stage: 98 °C for 15 s, 55–65 °C for 30 s, 72 °C for 3 min; 25 cycles; 72 °C for 4 min; one cycle). The resulting PCR products were digested (20 U *Dpn*I, 16 h, 37 °C), purified (PCR clean-up gel extraction kit, Macherey-Nagel), and transformed into *E. coli* BL21-Gold (DE3) for expression.

### 2.3. Expression of EGFP-10xAla-TEV-LCI in 96-Well Microtiter Plates

Each transformant was transferred into one well of a 96-well microtiter plate (MTP; flat-bottom, polystyrene). The generation of glycerol stocks and the cultivation procedure were performed as previously described [19]. Cell pellets were stored at −20 °C until use. *E. coli* BL21 (DE3) gold cells were resuspended in lysozyme (150 µL; 1.5 mg/mL, in 50 mM Tris/HCl buffer, pH 8.0) and incubated (1 h, 37 °C, 900 rpm, 70% humidity; Multitron Pro, Infors AG, Bottmingen, Switzerland), followed by centrifugation (3200× *g*, 30 min, 4 °C; Eppendorf centrifuge 5810 R, Eppendorf AG, Hamburg, Germany). Obtained supernatants were used directly for further characterization.

### 2.4. Expression of EGFP-10xAla-TEV-LCI in Flasks for Purification

Protein production of the generated variants EGFP, EGFP-LCI WT, EGFP-LCI Y29R, EGFP-LCI G35R, and EGFP-LCI Y29R/G35R was performed in *E. coli* BL21 (DE3) gold cells. Flask expression and subsequent affinity purification of the EGFP-LCI variants were performed as previously described [19].

### 2.5. Screening EGFP-10xAla-TEV-LCI for Improved Binding to Polypropylene in the Presence of Surfactant

The binding of LCI and its generated muteins towards polypropylene in the presence of the surfactant Triton X-100 was analyzed using the ABBA screening system [17]. EGFP-LCI containing cell lysates (10 µL/well) were supplemented into 90 µL/well Tris/HCl buffer (pH 8.0, 50 mM) and incubated (10 min, room temperature, 600 rpm; MTP shaker, TiMix5, Edmund Bühler GmbH, Hechingen, Germany) in black PP-MTP (flat bottom). The MTP wells were washed with Tris/HCl buffer (100 µL/well; pH 8.0, 50 mM, 5 min, room temperature, 600 rpm) in two subsequent washing steps, to avoid unspecific binding of other lysate proteins. In the final step, the surfactant Triton X-100 (100 µL/well, 1 mM) was supplemented and incubated (5 min, room temperature, 600 rpm). After removal of the liquid, bound EGFP-anchor peptides were detected directly on the PP surface with the 96-well MTP reader FLUOstar Omega (BMG LABTECH GmbH, Ortenberg, Germany) (excitation (ex.) 485 nm, emission (em.) 520 nm, gain 1000, 35 reads/well). The fluorescence of bound-identified variants was compared to the fluorescence of the bound EGFP-LCI wild type. The background fluorescence of empty wells (41.1 ± 5.7 RFU) were determined for each measurement and subtracted from the fluorescence values of the variants and the wild type. Subsequently, values of the variants were divided by the wild type values (105.0 ± 14.9 RFU). Clones were considered as binding (active) variants, when the binding signal was 0.8-fold or higher compared to the wild type. Variants with a variant–wild type (V/WT) ratio of >2 were considered as improved binding peptide variants.

### 2.6. Computational Analysis

The initial coordinates were taken from the solution NMR structure of LCI (PDB ID: 2B9K [23]). 2B9K contains 21 conformers. The 3D structure for analysis was based on conformer 3. The hydrophobic surface area of LCI was visualized (hydrophobic surface area corresponds to grey/hydrophilic surface area corresponds to blue) using Discovery Studio Client, Release 4.0 (Accelrys Software http://accelrys.com/).

### 2.7. Characterization of Purified EGFP-10xAla-TEV-LCI-Binding to Polypropylene in the Presence of Surfactant

The polymer anchor peptide-binding assay was also used to characterize the binding kinetics of purified EGFP-LCI and identified variants towards polypropylene with and without the surfactant Triton X-100. In the binding step, purified EGFP-LCI and EGFP-LCI KR-2 variant (100 µL, 0–2.5 µM) were incubated (10 min, room temperature, 600 rpm) in black PP-MTP (flat bottom). The MTP wells were washed with Tris/HCl buffer (100 µL/well; pH 8.0, 50 mM, 5 min, room temperature, 600 rpm) in three subsequent washing steps. In the final desorption step, either buffer (100 µL/well; pH 8.0, 50 mM) or non-ionic surfactant Triton X-100 (1 mM, pH 8.0) was supplemented into the wells and incubated (5 min, room temperature, 600 rpm). The liquid was removed, and Tris/HCl buffer (100 µL/well; pH 8.0, 50 mM) was supplemented into the wells. The binding was detected with the 96-well MTP reader FLUOstar Omega (ex. 485 nm, em. 520 nm, gain 1000, 35 reads/well).

The effect of the Triton X-100 concentration on peptide binding was investigated in a similar manner. In the binding step, EGFP-LCI and EGFP-LCI KR-2 variant (20 µL, 10 µM) were supplemented into Tris/HCl buffer (80 µL/well; pH 8.0, 50 mM). Incubation and washing steps were performed as stated above. In the final desorption step, 100 µL/well Triton X-100 (0–10 mM) were supplemented and incubated (5 min, room temperature, 600 rpm). The liquid was removed, and Tris/HCl buffer (100 µL/well; pH 8.0, 50 mM) was supplemented into the wells. The binding was detected with the 96-well MTP reader FLUOstar Omega (ex. 485 nm, em. 520 nm, gain 1000, 35 reads/well).

## 3. Results

The results section is divided into two parts. The first part describes the KnowVolution campaign with its four phases. Phase I consists of the generation and screening of an epPCR library of LCI. Phase II consists of the generation and screening of 11 SSM libraries of potentially beneficial positions that have been identified in phase I. Phase III describes the computational analysis of the identified beneficial amino acid substitutions. Phase IV consists of the recombination of beneficial amino acid substitutions, which finally resulted in the generation of the variant EGFP-LCI KR-2. The second part describes the characterization of the identified LCI variants in comparison to the wild type with respect to binding strengths on polypropylene in the presence of Triton X-100.

### 3.1. KnowVolution of the Anchor Peptide LCI for Improving Polypropylene-Binding Strength

**Figure 1:**
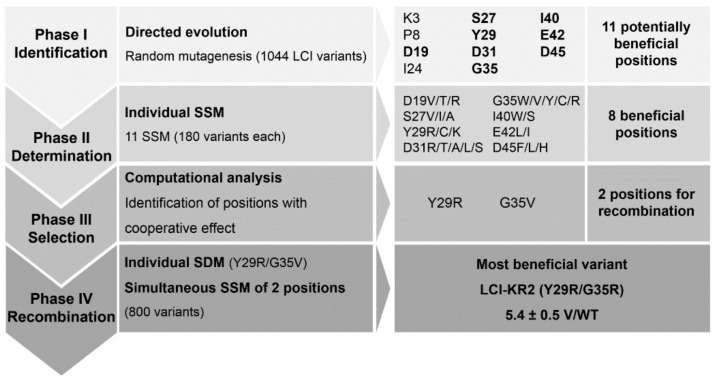
Overview of the knowledge-gaining directed evolution (KnowVolution) strategy of the polypropylene anchor peptide campaign. The campaign was performed to increase the binding strength of liquid chromatography peak I peptide, 47 aa (LCI ) toward polypropylene in the presence of the non-ionic surfactant Triton X-100. Phase I: a random mutagenesis library was generated and screened and yielded 11 potentially beneficial positions (K3, P8, D19, I24, S27, Y29, D31, G35, I40, E42, and D45). Phase II: each position was saturated individually (site-saturation mutagenesis (SSM), using NNK-codon) and two 96-well microtiter plates (MTPs) were screened per position. Phase III: the identified beneficial positions and substitutions were analyzed computationally to group beneficial positions for exploiting cooperative effects. Phase IV: positions Y29 and G35 were recombined by site-directed mutagenesis (SDM), yielding the variant LCI-Y29R/G35V and by simultaneous SSM, yielding variant KR-2 (Y29R/G35R).

Figure 1 shows an overview of the performed KnowVolution campaign identifying 11 potential positions in phase I from which 8 really contributed to increased binding strength. Finally, through recombination and computational-assisted analysis the variant LCI Y29R G35R was generated with a >5-fold improved binding in the presentence of Triton X-100.

**Phase I: Identification.** Random mutagenesis was performed to identify key positions or regions, which influence the binding of LCI to PP. The random mutagenesis library was generated with error-prone PCR (epPCR, 0.8 mM MnCl_2_) [32], and 35% of the generated LCI variants were capable of binding to PP in the presence of 1 mM Triton X-100 [17]. In total, 1044 clones were screened for stronger PP-binding in a 96-well MTP format with the recently reported ABBA screening system [17].

The screening of the epPCR library yielded ten epLCI variants with stronger binding to PP. EpLCI variants with a variant–wild type (V/WT) ratio ≥2.0 were considered as stronger PP-binders. The best-identified variant LCI-M3-PP resulted in a V/WT ratio of 4.1 ± 0.5 carrying three amino acid substitutions. Variants LCI-M1-PP and LCI-M2-PP resulted in V/WT ratios of 3.4 ± 0.8 and 2.5 ± 0.2, respectively, and were previously reported [17]. Potential key positions are defined as positions that were substituted in two or more improved variants. Sequencing results of the identified epLCI variants revealed 11 potential key positions (K3, P8, D19, I24, S27, Y29, D31, G35, I40, E42, and D45), which affect the PP-binding of LCI. The binding improvements and the sequencing results for the identified random mutagenesis variants are summarized in Table S2.

**Figure 2:**
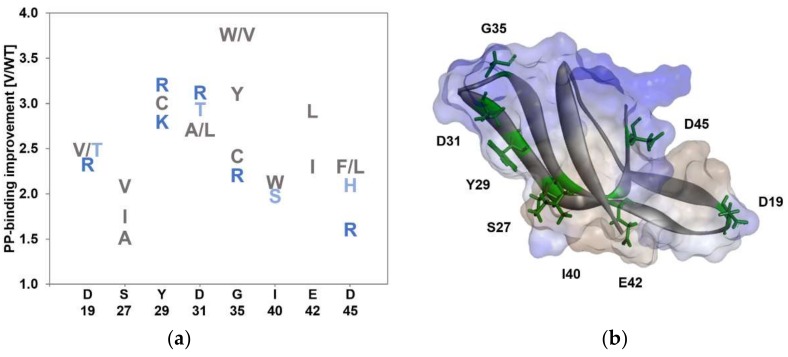
Amino acid substitutions at LCI key positions for stronger PP-binding: (**a**) Beneficial amino acid substitutions for selection with non-ionic surfactant Triton X-100 (identified by site-saturation mutagenesis). Positions with substitutions that led to variants with a variant–wild type (V/WT) ratio ≥1.5 were considered as beneficial key positions. Amino acids are colored according to their chemical properties (positively charged: K, and R (dark blue); polar: H, S, T, N, and Q (light blue); hydrophobic: F, Y, W, A, V, L, I, and C (grey); (**b**) Model of LCI wild type generated with the Discovery Studio 4.0 Client. Identified beneficial positions (D19, S27, Y29, D31, G35, I40, E42, and D45) are highlighted in green. Blue indicates hydrophilic surface areas, and grey scales indicate hydrophobic surface areas. The model is based on PDB entry 2B9K conformer 3 [23].

**Phase II: Determination.** All 11 potential key positions were subjected to individual SSM in order to identify the most advantageous substitutions at each position with respect to stronger PP-binding. Binding improvement and sequencing results of each SSM library are summarized in Table S3; the substitutions at identified key positions and their corresponding improvements are summarized in Figure 2.

SSM library variants with a variant–wild type (V/WT) ratio ≥1.5 were considered as stronger PP-binders. The V/WT limit was lowered to 1.5 in this phase to gain as much information as possible about the individual positions. The saturation library of position G35 yielded the strongest polypropylene-binding variant G35V with a 3.8 ± 0.5-fold stronger binding compared with the LCI wild type. The saturation of positions D19, I24, S27, Y29, D31, G35, I40, E42, and D45 led to the identification of variants with a >1.5-fold stronger PP-binding. The saturation libraries of the positions K3, P8, and I24 did not yield any improved variants (V/WT ratio below 1.5).

Figure 2a shows the most beneficial substitutions at each of the identified key positions (D19, I24, S27, Y29, D31, G35, I40, E42, and D45). The substitutions are colored according to their hydrophobicity and charge (hydrophobic aa = grey, polar aa = light blue, charged aa = dark blue). Notably, all negatively charged amino acids in the LCI wild type sequence (D19, D31, E42, and D45; colored in blue) were substituted to improve the binding on PP surfaces.

**Phase III: Selection.** The identified beneficial key positions for stronger PP-binding were visualized by Discovery Studio 4.0 Client using PDB file 2B9K (Figure 2b). The aim was to investigate their proximity to each other and the chemical nature of the amino acid substitutions (hydrophobic; grey; hydrophilic blue). Interestingly, the positions S27, Y29, D31, and G35 cluster in the turn 2 (between β3 and β4 strands; Figure 2b). Y29 is part of the hydrophobic peptide surface, while G35 lies in the hydrophilic region. The libraries Y29 and G35 resulted in the best binding variants (Y29R and G35V). To investigate the cooperative effect of the identified beneficial amino acid substitutions variants EGFP-LCI Y29R/G35V, EGFP-LCI E42L/D45F, EGFP-LCI Y29R/E42L, EGFP-LCI D19T/Y29R, EGFP-LCI D19T/E42L, and EGFP-LCI S27I/E42L were generated.

**Figure 3:**
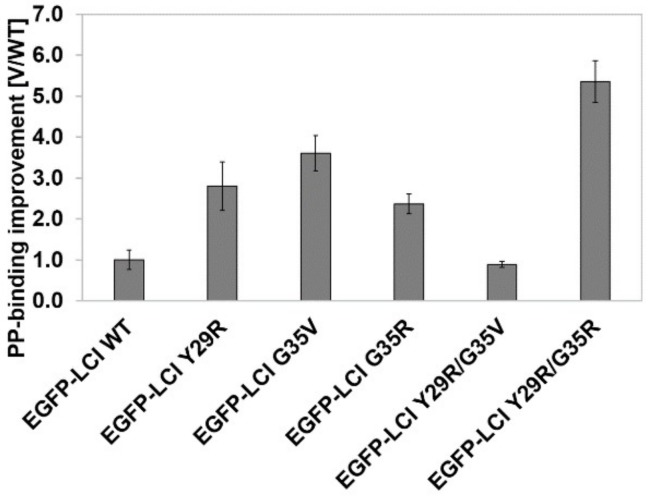
Binding performance of KnowVolution variants of the (polypropylene) PP-binding peptide LCI. Single substituted variants EGFP-LCI Y29R, EGFP-LCI G35V, EGFP-LCI G35R and recombination variant EGFP-LCI KR-2 (Y29R/G35R) were compared in PP-binding strength. Variant–wild type (V/WT) ratios were determined under screening conditions (selection with 1 mM Triton X-100).

**Phase IV: Recombination.** Recombination proved to be a challenging task: the expression levels of the recombined LCI-muteins (EGFP-LCI Y29R/G35V, EGFP-LCI E42L/D45F, EGFP-LCI D19T/E42L, and EGFP-LCI S27I/E42L; Figure S1) were barely sufficient for SDS-gel detection. Also, the recombination variant EGFP-LCI Y29R/G35V (generated by SDM) of the two most beneficial substitutions was weakly expressed in the used *E. coli* expression system; furthermore, no improved binding strength was obtained in comparison with the single substitute LCI-variants (Figure S1). This indicates that the LCI fold cannot tolerate two or more substitutions. We therefore investigated cooperative effect by simultaneous saturation of the two positions that provided the best binding variants in phase II (Y29 and G35). Subsequently, a simultaneous SSM at the positions Y29 and G35 was performed to investigate whether the simultaneous exchange would yield a different amino acid combination with further improved binding strength. A reduced triplet set (MVW) was used for diversity generation at position Y29 (charged substitutions were predominantly observed to be beneficial). A NNK triplet was used for position G35 to obtain full diversity. The library contains 384 different LCI variants, and 800 LCI variants were screened. The screening system for polypropylene-binding yielded variant EGFP-LCI Y29R/G35R (EGFP-LCI KR-2) with an improvement of 5.4 ± 0.5 (V/WT ratio, Figure 3). The single substitutions Y29R and G35R were individually observed in phase II with improvements of 2.8 ± 0.6 and 2.4 ± 0.2 (V/WT ratio), respectively. A comparison of the loss of binding strength in the recombination variant EGFP-LCI Y29R/G35V and the stronger binding of variant EGFP-LCI Y29R/G35R indicates the importance of cooperative interactions.

### 3.2. Characterization of EGFP-10xAla-TEV-LCI and EGFP-10xAla-TEV-LCI KR-2-Binding to Polypropylene in the Presence of Triton X-100

**Figure 4:**
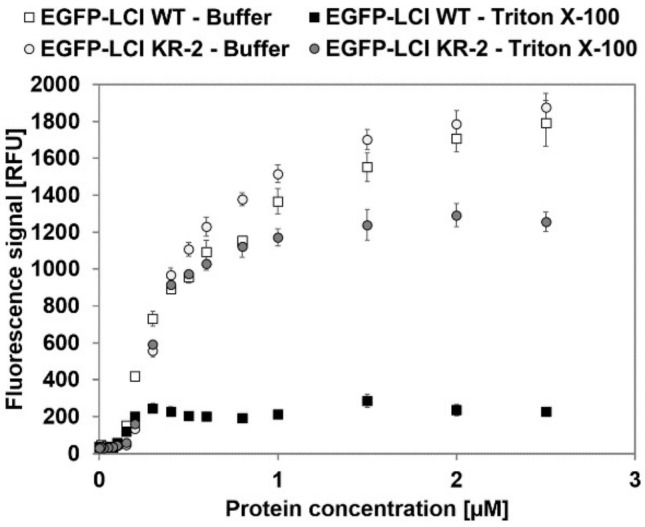
Determination of EGFP-LCI WT and EGFP-LCI KR-2 equilibrium dissociation constant K_D_ and maximal binding capacity *B*_Max_. The binding saturation of EGFP-LCI WT and EGFP-LCI KR-2 was determined in a PP-MTP well (contact area 35 mm^2^) after 3 cycles of washing with Tris/HCl buffer (pH 8.0, 50 mM) and elution with either Tris/HCl buffer (pH 8.0, 50 mM) or Triton X-100 (pH 8.0, 1 mM). Open square: EGFP-LCI WT eluted with buffer; black square: EGFP-LCI WT eluted with Triton X-100; open dot: EGFP-LCI KR-2 eluted with buffer; and grey dot: EGFP-LCI KR-2 eluted with Triton X-100 (pH 8.0, 1 mM).

Binding constants (equilibrium dissociation constant *K*_D_ and maximal binding capacity *B*_Max_) of EGFP-LCI WT and EGFP-LCI Y29R/G35R with and without Triton X-100 were determined using the established MTP-binding assay (contact area 35 mm^2^; Figure 4). The influence of introduced mutation on EGFP fluorescence was investigated. The results showed no significant difference in fluorescence in the investigated range (Figure S2). PP-binding was characterized using purified LCI and LCI-muteins to avoid unspecific interactions with proteins present in the cell lysate.

The obtained fluorescence signals were correlated to the corresponding protein amounts using a fluorescence calibration curve (Figure S3), and the results were fitted with a one-site binding model using GraphPad Prism software V6.01 to obtain *K*_D_ and *B*_Max_ (Table 1). The coefficient of determination (*R*^2^) indicates the quality of the fit.

EGFP-LCI KR-2 and EGFP-LCI have almost the same maximal binding capacity and dissociation constant when no surfactant is present in the elution buffer. When Triton X-100 is supplemented in the elution buffer, the maximal binding capacity of EGFP-LCI WT is decreased by 7.9-fold, and the dissociation constant is decreased by 2.6-fold when compared with the elution with buffer. EGFP-LCI KR-2, however, resulted in a 4.8-fold increased maximal binding capacity when compared to EGFP-LCI WT. The dissociation constant of EGFP-LCI KR-2 is 1.8-fold higher than that of the wild type, which indicates a decrease in binding affinity for PP through the introduced substitutions. However, EGFP-LCI KR-2 results in a coating with 7.0 pmol (4.2155 × 10^12^ molecules) more protein per cm^2^ than that of the wild type. The PP-binding strength of purified EGFP-LCI WT and EGFP-LCI KR-2 was determined in the presence of Triton X-100 (Figure S4). Both EGFP-LCI WT and EGFP-LCI KR-2 showed a stable fluorescent signal of 1245 RFU and 1509 RFU, respectively (after wash with an elution buffer containing 0–0.005 mM Triton X-100). The EGFP-LCI WT binding signal is stable for elution from 0.1–10 mM Triton X-100 and corresponds to 1.1 pmol/cm^2^ bound EGFP-LCI. The EGFP-LCI KR-2 binding signal increased at concentrations above 0.1 mM Triton X-100 and reached a plateau at ~1010 RFU (7.6 pmol/cm^2^ bound LCI variant).

## 4. Discussion

PBPs have a huge potential for the directed immobilization of proteins (e.g., enzymes, bioactive peptides, or antigens onto all kind of surfaces). Bio-functionalization of surfaces in products requires a binding performance that is guaranteed under application conditions. For example, on textiles the binding must be ensured even when the functionalized textile material is washed with commercial laundry detergents containing non-ionic and anionic surfactants. Previous reports showed that such a stability to surfactants is not inherent in PBPs [19], but can be improved through protein engineering [17]. In order to ensure an efficient KnowVolution campaign, the concentration of non-ionic surfactant Triton X-100 was chosen to be 1 mM (see Figure S4). Insights into the molecular mode of interaction between the peptide, surface, and surfactants is a prerequisite for a rational design of surfactant-tolerant PBPs. The performed KnowVolution campaign for the binding peptide LCI toward improved PP-binding in the presence of Triton X-100 yielded 8 key positions (D19, S27, Y29, D31, G35, I40, E42, and D45) that govern interactions with the hydrophobic PP surface. Positions S27, Y29, D31, G35, and I40 are part of the turn 2 in which the key positions face the same direction (Figure 2b; towards the PP surface). Therefore, it is highly probable that side chains at these five positions interact directly with the PP surface. As a general trend, one could observe that four out of the five positions in the turn included amino acid substitutions for hydrophobic amino acids (S27A/I/V, D31A/L, G35V/W/Y, and I40W), which likely foster hydrophobic interactions with the PP surface. As expected, all negatively charged amino acids in the wild type sequence of LCI (D19, D31, E42, and D45) were substituted in the KnowVolution campaign (phase I, Table S2). Unexpected was the general trend of an increased number of substitutions for positively charged amino acids at positions (D19, Y29, D31, G35, and D45), which was also previously reported [33,34] without providing an explanation. In fact, the existence of a positive charge at the interface may destructure water molecules induced in hydrophobic hydration and consequently increase hydrophobic interactions [26].

The “most beneficial” variant EGFP-LCI KR-2 (EGFP-LCI Y29R/G35R) harbors two substitutions for positively charged amino acids (Figure 2b), and the grand average of hydropathy (GRAVY) was reduced from −0.351 to −0.506. The latter indicates an increase in hydrophilicity, through the introduction of arginines into the peptide sequence.

Positively charged amino acid residues arginine and lysine were identified as essential for binding to other material surfaces, such as TiO_2_, Cu_2_O, silica, or gold [11,35,36,37], but their role in the binding “mechanism” to uncharged surfaces remains unclear [11]. Tsai et al. suggested that solvent interactions (cosolvent acetonitrile) play a major role in peptide adsorption to hydrophobic surfaces. Peptides with higher electro-positivity were postulated to bind “less” solvent and therefore adsorbed preferably on the material surface [38].

The recombination of the identified substitutions proved to be very challenging, as many of the generated recombination variants (five out of six LCI recombinants) lacked overexpression, or the binding strength did not improve when compared with the single substituted variants. The latter indicates that the LCI fold (containing a four-strand antiparallel β-sheet) with a total of only 47 amino acids cannot (to a large extent) tolerate two or more substitutions. Therefore, to simultaneously saturate two positions and to screen for strongly binding variants proved to be a better strategy than recombination by site-directed mutagenesis. State of the art methods in directed evolution, such as the OmniChange technology, allow us to simultaneously saturate up to five positions [39].

The LCI KnowVolution campaign finally resulted in the generation of the variant EGFP-LCI KR-2 with an improved binding capacity of 8.84 ± 0.05 pmol/cm^2^ in the presence of 1 mM Triton X-100. A comparison of adhesion promotors to hydrophilic surfaces show that the PP-coating with EGFP-LCI KR-2 reaches densities of 550 ng/cm^2^ (Buffer) and 320 ng/cm^2^ (Triton X-100) that have so far only been reported for hydrophilic surfaces. In detail, reported plastic-binding peptides with the fusion partner glutathione S-transferase (GST) resulted in binding densities between 580 ng/cm^2^ (PS-19-6 on hydrophilic PS) and 260 ng/cm^2^ (OMP6 on polycarbonate) [40].

In conclusion, the KnowVolution strategy was used for the first time to improve the binding strength of a polymer-binding peptide through a directed evolution campaign. In the process, 8 key positions for improvement in PP-binding were identified and enabled to fine tune binding strength through computational-assisted design. As general trend, one could observe that negative charges were removed from the LCI wild type to improve binding strength, and surprisingly positive charges seem to contribute to stronger binding strength (EGFP-LCI KR-2). These results are, at first glance, contradictory and require additional computational studies to understand the role that water binding to LCI plays in modulating the interactions with the hydrophobic PP surface. Additionally, more efficient recombination strategies are required because many double substituted variants lost their improved binding strength or were not expressible (the main lesson learned). Therefore, a deeper molecular understanding by computational modeling is required to fully explore the potential of protein engineering (directed evolution) in boosting surface-binding strength.

## Figures and Tables

**Table 1:** Equilibrium dissociation constant *K*_D_ and maximal binding capacity *B*_Max_ for the binding of EGFP-LCI WT and EGFP-LCI KR-2 to PP.

Variant	Elution	*B*_Max_ [pmol/cm^2^]	*K*_D_ [µM]	*R*^2^
EGFP-LCI WT	Buffer	14.63 ± 0.30	0.47 ± 0.02	0.985
	Triton X-100	1.86 ± 0.06	0.18 ± 0.02	0.910
EGFP-LCI KR-2	Buffer	15.19± 0.56	0.54 ± 0.04	0.980
	Triton X-100	8.84 ± 0.05	0.34 ± 0.03	0.997

## Supplementary Materials

The following are available online at http://www.mdpi.com/2073-4360/10/4/423/s1, Table S1: Primer sequences, Table S2: Summary of binding performance and amino acid substitutions found in improved EGFP-epLCI variants screened for improved PP-binding in the presence of 1 mM Triton X-100, Table S3: LCI key positions and identified amino acid substitutions for improved PP-binding, Figure S1: Expression and performance of PP-binding peptide LCI variants, Figure S2: Fluorescence of EGFP (grey), EGFP-LCI (white), and EGFP-LCI KR-2 in the protein concentration range of 0.001–0.25 µM, Figure S3: Quantification of fluorescence intensity of EGFP-LCI (concentrations: 0–0.06 µM), Figure S4. PP-binding of EGFP-LCI WT (white) and EGFP-LCI KR-2 (grey) after selection with nonionic surfactant Triton X-100 (pH 8.0, 0.0001–10 mM).
