# Supplementary material for: KnowVolution of the Polymer-Binding Peptide LCI for Improved Polypropylene Binding
Source: Polymers (Basel). 2018 Apr 10;10(4):423. doi: 10.3390/polym10040423 (PMC6415234; doi:10.3390/polym10040423)
Supplement: Supplementary file 1 [file polymers-10-00423-s001.pdf]

Supporting information

KnowVolution of the polymer binding peptide LCI for polypropylene binding

Kristin Rübsam[1], Mehdi D. Davari [1], Felix Jakob[2], Ulrich Schwaneberg[1,2*]

[1] RWTH Aachen University, Worringerweg 3, D-52074 Aachen, Germany; k.ruebsam@biotec.rwth-aachen.de (K.R.); u.schwaneberg@biotec.rwth-aachen.de (U.S.)

[2] DWI - Leibniz-Institute for Interactive Materials, Forckenbeckstrasse 50 D-52074 Aachen, Germany; jakob@dwi.rwth-aachen.de (F.J.)

* Correspondence: u.schwaneberg@biotec.rwth-aachen.de; Tel.: +49 241 80 24170

Academic Editor: name
Received: date; Accepted: date; Published: date

Table S1. Primer sequences.

Primer	Sequence 5′ → 3′
F-epLCI	GCCGAAAATCTGTATTTTCAGGGT
R-epLCI	GTCGACGGAGCTCGAATTCTTA
F-K3-SSM	GTTGCGCCATTNNKCTGGTTCAGAG
R-K3-SSM	CTCTGAACCAGMNNAATGGCGCAAC
F-P8-SSM	CTGGTTCAGAGCNNKAATGGTAATTTTGCAGC
R-P8-SSM	GCTGCAAAATTACCATTMNNGCTCTGAACCAG
F-N9-SSM	CTGGTTCAGAGCCCGNNKGGTAATTTTGCAGC
R-N9-SSM	GCTGCAAAATTACCMNNCGGGCTCTGAACCAG
F-D19-SSM	GCAGCAAGCTTTGTTCTGNNKGGCACCAAATGG
R-D19-SSM	CCATTTGGTGCCMNNCAGAACAAAGCTTGCTGC
F-I24-SSM	GGATGGCACCAAATGGNNKTTCAAAAGC
R-I24-SSM	GCTTTTGAAMNNCCATTTGGTGCCATCC
F-Y29-SSM	CTTCAAAAGCAAANNKTATGACAGCAGC
R-Y29-SSM	GCTGCTGTCATAMNNTTTGCTTTTGAAG
F-D31-SSM	GCAAATACTATNNKAGCAGCAAAGGTTATTGGGTGGGT
R-D31-SSM	ACCCACCCAATAACCTTTGCTGCTMNNATAGTATTTGC
F-S33-SSM	GCAAATACTATGACAGCNNKAAAGGTTATTGGGTGGGT
R-S33-SSM	ACCCACCCAATAACCTTTMNNGCTGTCATAGTATTTGC
F-G35-SSM	CTATGACAGCAGCAAANNKTATTGGGTGGGT
R-G35-SSM	ACCCACCCAATAMNNTTTGCTGCTGTCATAG
F-I40-SSM	TGGGTGGGTNNKTATGAAGTGTGG
R-I40-SSM	CCACACTTCATAMNNACCCACCCA
F-E42-SSM	TGGGTGGGTATTTATNNKGTGTGGGATCGC
R-E42-SSM	GCGATCCCACACMNNATAAATACCCACCCA
F-W44-SSM	GTATTTATGAAGTGNNKGATCGCAAATAAG
R-W44-SSM	CTTATTTGCGATCMNNCACTTCATAAATAC
F-D45-SSM	GAAGTGTGGNNKCGCAAATAAGAATTCGAGCTCCG
R-D45-SSM	CGGAGCTCGAATTCTTATTTGCGMNNCCACACTTC
F-Y29R	CTTCAAAAGCAAACGTTATGACAGCAGC
R-Y29R	GCTGCTGTCATAACGTTTGCTTTTGAAG
F-G35V	CTATGACAGCAGCAAAGTGTATTGGGTGGGT
R-G35V	ACCCACCCAATACACTTTGCTGCTGTCATAG
F-Y29/G35	CTTCAAAAGCAAAMVWTATGACAGCAGCAAANNKTATTGGGTGGGT
R-Y29/G35	ATACCCACCCAATAMNNTTTGCTGCTGTCATAWBKTTTGCTTTTGAAG

Table S2. Summary of binding performance and amino acid substitutions found in improved EGFP-epLCI variants screened for improved PP binding in presence of 1 mM Triton X-100. Potential beneficial positions are underlined.

Variant	V/WT	Substitutions
LCI-M1-PP	3.4 ± 0.8	I24T Y29H E42K
LCI-M2-PP	2.5 ± 0.2	D31V E42G
LCI-M3-PP	4.1 ± 0.5	D31V S32C D45V
LCI-M4-PP	3.6 ± 0.2	K3R P8Q N9K G10C D19G I24T S27G G35D W44R D45V
LCI-M5-PP	2.9 ± 0.4	Q6H Y29F I40T D45A
LCI-M6-PP	2.6 ± 0.2	P8L S15R S27C D45G
LCI-M7-PP	2.6 ± 0.8	W23R S33T Y36C
LCI-M8-PP	2.6 ± 0.4	L4Q K34R E42V
LCI-M9-PP	2.6 ± 0.3	I2F K3Q N11S D19G G35C K47R
LCI-M10-PP	2.5 ± 0.1	F16L I24S I40T D45Y

Table S3. LCI key positions and identified amino acid substitutions for improved PP binding.

Position	Variant	V/WT
K3	K3W	1.3 ± 0.1
P8	P8R	1.6 ± 0.3
D19	D19V	2.5 ± 0.5
	D19T	2.5 ± 0.5
	D19R	2.4 ± 0.4
I24	I24G	1.4 ± 0.1
	I24L	1.3 ± 0.2
S27	S27V	2.1 ± 0.2
	S27I	1.7 ± 0.2
	S27A	1.5 ± 0.2
Y29	Y29R	3.2 ± 0.5
	Y29C	3.0 ± 0.2
	Y29K	2.8 ± 0.4
D31	D31R	3.1 ± 0.4
	D31T	3.0 ± 0.3
	D31A	2.9 ± 0.1
	D31L	2.9 ± 0.2
	D31S	2.7 ± 0.3
G35	G35W	3.8 ± 0.5
	G35V	3.7 ± 0.4
	G35Y	3.1 ± 0.4
	G35C	2.4 ± 0.3
	G35R	2.2 ± 0.2
I40	I40W	2.1 ± 0.7
	I40S	2.0 ± 0.4
E42	E42L	2.9 ± 0.5
	E42I	2.3 ± 0.3
D45	D45F	2.3 ± 0.3
	D45L	2.3 ± 0.2
	D45H	2.1 ± 0.3

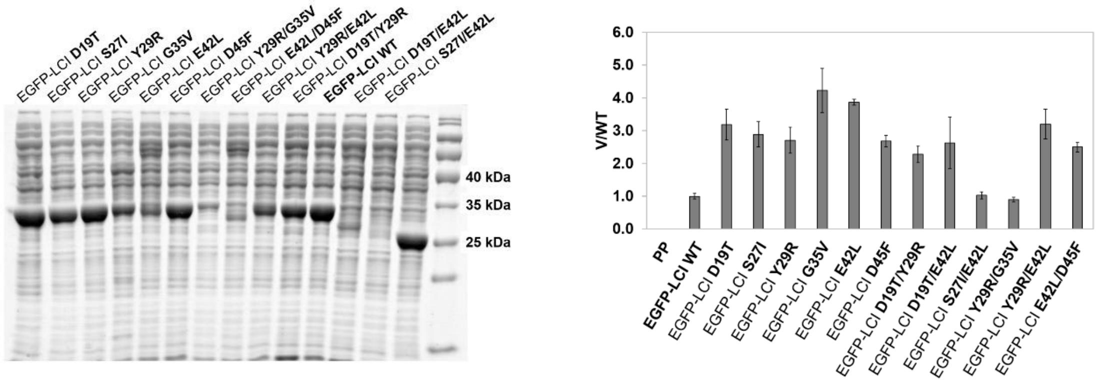

(a) **(b)**

Figure S1. Expression and performance of PP-binding peptide LCI variants. SSM variants LCI Y29R and LCI G35V and generated recombination variants LCI Y29R/G35V were produced in MTP and resulting CFE was used for SDS-PAGE to evaluate expression level (a) and in ABBA screening system to evaluate binding performance (b).

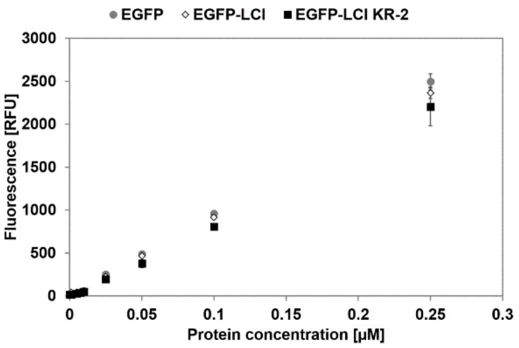

Figure S2. Fluorescence of EGFP (grey), EGFP-LCI (white), and EGFP-LCI KR-2 in the protein concentration range of 0.001-0.25 µM. The fluorescence was detected with 96-well MTP reader FLUOstar Omega (BMG LABTECH GmbH, Ortenberg, Germany) (excitation (ex.) 485 nm, emission (em.) 520 nm, gain 750, 35 reads/well).

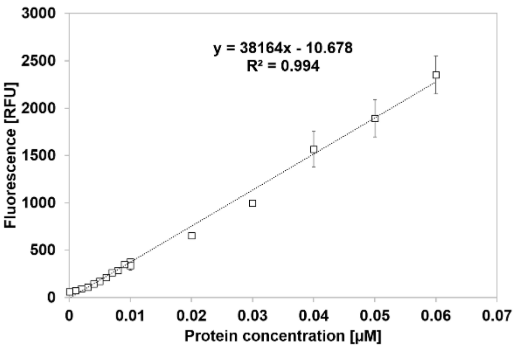

Figure S3. Quantification of fluorescence intensity of EGFP-LCI (concentrations: 0-0.06 µM). Detection was performed with FLUOstar Omega (exc. 485 nm, em. 520 nm, gain 1000). Each concentration was determined in triplicates. Error bars indicate the standard deviation.

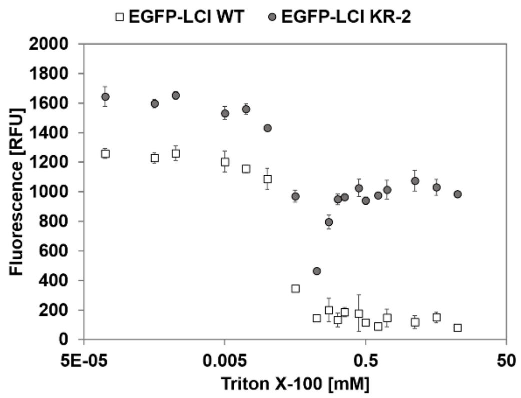

Figure S4. PP-binding of EGFP-LCI WT (white) and EGFP-LCI KR-2 (grey) after selection with nonionic surfactant Triton X-100 (pH 8.0, 0.0001-10 mM).
